# Natural Products as Source of Potential Dengue Antivirals

**DOI:** 10.3390/molecules19068151

**Published:** 2014-06-17

**Authors:** Róbson Ricardo Teixeira, Wagner Luiz Pereira, Ana Flávia Costa da Silveira Oliveira, Adalberto Manoel da Silva, André Silva de Oliveira, Milene Lopes da Silva, Cynthia Cânedo da Silva, Sérgio Oliveira de Paula

**Affiliations:** 1 Departamento de Química, Universidade Federal de Viçosa, 36570-900 Viçosa, MG, Brazil; 2 Instituto Federal de Educação, Ciência e Tecnologia do Norte de Minas, 39900-000 Almenara, MG, Brazil; 3 Departamento de Microbiologia, Universidade Federal de Viçosa, 36570-900 Viçosa, MG, Brazil; 4 Departamento de Biologia Geral, Universidade Federal de Viçosa, 36570-900 Viçosa, MG, Brazil

**Keywords:** dengue virus, dengue fever, antiviral natural products

## Abstract

Dengue is a neglected disease responsible for 22,000 deaths each year in areas where it is endemic. To date, there is no clinically approved dengue vaccine or antiviral for human beings, even though there have been great efforts to accomplish these goals. Several approaches have been used in the search for dengue antivirals such as screening of compounds against dengue virus enzymes and structure-based computational discovery. During the last decades, researchers have turned their attention to nature, trying to identify compounds that can be used as dengue antivirals. Nature represents a vast reservoir of substances that can be explored with the aim of discovering new leads that can be either used directly as pharmaceuticals or can serve as lead structures that can be optimized towards the development of new antiviral agents against dengue. In this review we describe an assortment of natural products that have been reported as possessing dengue antiviral activity. The natural products are organized into classes of substances. When appropriate, structure-activity relationships are outlined. The biological assays used to assess antiviral activity are briefly described.

## 1. Introduction

Dengue fever (DF) and dengue hemorrhagic fever (DHF) are acute febrile diseases transmitted by mosquitoes. Nowadays, they are the most rapidly spreading mosquito-borne diseases in the world. About 2.5 billion people, two-fifths of the world’s population, are now at risk of infection and 50 million cases of DF are reported worldwide every year [1]. In recent decades, these diseases have spread to over more than 100 countries [2]. The World Health Organization (WHO) estimates that the annual global incidence of dengue is close to 390 million, a number nearly three times higher than the number of cases estimated by the same organization for 2009 [3].

Increased urbanization along with substandard housing, unreliable water supply, and poor sanitation provide a suitable environment for vector proliferation in close proximity to human hosts. Dengue proliferation became even more problematic in the Americas due to the collapse of vector eradication programs in the 1970s [4].

The dengue virus particle is about 50 nm in diameter. The 10,723-nucleotide RNA genome encodes an uninterrupted open reading frame (ORF), directing the synthesis of a polyprotein precursor in the order NH_2_-C-prM-E-NS1-NS2A-NS2B-NS3-NS4A-NS4B-NS5-COOH, where C is the capsid protein, M is the membrane-associated protein, E is the envelope protein, and NS1 through NS5 are nonstructural proteins [5].

The disease has four viral serotypes (DENV 1–4), and its spectrum ranges from asymptomatic infection to dengue fever (DF), dengue hemorrhagic fever (DHF), and dengue shock syndrome (DSS), and may lead to patient death [6]. All four serotypes of dengue virus are transmitted to humans by the *Aedes aegypti* and *Aedes albopictus* mosquitoes [7].

During a dengue outbreak that struck Malaysia’s Sarawak state, on Borneo, blood and serum samples from a severe case labeled “dengue 4” were collected, and later, the sequence of the genome showed that the virus occupies a new branch on the dengue family tree, suggesting the serotype DENV-5 [3]. Provided that this new serotype is transmissible as DENV 1–4, it might follow a similar pattern of geographical spread as described by Messina and co-workers. By using several maps, the authors demonstrated the expansion of the serotypes throughout the world, the growth of hyperendemicity (coexistence of multiple serotypes), and the establishment of dengue as an important infectious disease of global public health importance [8].

To date, there is no clinically approved dengue vaccine or antiviral for humans, even though there have been great efforts towards this end. The treatment of the disease is limited to supportive care [9,10] with analgesics, fluid replacement and bed rest [11]. Aspirin, non-steroidal anti-inflammatory drugs (NSAIDs), and corticosteroids should be avoided. Special attention should be given to severe cases of dengue in terms of fluid administration and treatment of hemorrhage. A placebo-controlled, double-blind investigation was conducted with sixty-three children having severe dengue shock syndrome in two hospitals in Thailand. The children were completely randomized into two groups. One of the groups was treated with a single dose of steroidal drug methylprednisolone and a placebo was administered to the other group. The study revealed that there was no significant difference in mortality between the groups [12].

The cost of dengue to society is considerable, from lost wages and diminished productivity to costs related to care-giving and direct medical expenses. The cost of dengue in the Western Hemisphere alone is estimated to be US$2.1 billion per year [13]. In view of these problems, an efficient dengue vaccine or antiviral is highly desirable.

Different approaches have been used in the search for dengue antivirals, such as screening of compounds against dengue enzymes [14] and structure-based computational discovery [15]. During the last decades, researchers have turned their attention to nature, trying to identify compounds that can be used as dengue antivirals. In fact, nature is a fantastic reservoir of substances that can be used directly as pharmaceuticals or can serve as lead structures that can be optimized towards the development of new therapeutic agents [16,17,18,19,20,21,22,23].

Several plants around the world present potential dengue antiviral activity. Recently, Kadir and co-workers reviewed sixty nine studies from 1997 to 2012 related to plants presenting potential antidengue activity [24]. It should be mentioned that according to a WHO factsheet, 80% of the population in some Asian and African countries depends on traditional medicine as their primary health care due to economic and geographical constraints [25,26,27]. In view of their few (or lack of) adverse effetcts, the world-wide use of medicinal plants or herbal-based medicine is steadily growing.

Even though a number of plants are known for their antidengue activity, few investigations have been published related to isolation (identification) of compounds from plants and subsequent evaluation of their dengue antiviral activities. We describe in this review several investigations which resulted in the isolation (or identification) of compounds endowed with antiviral dengue activity, most of them isolated from plants. The review also covers metabolites isolated from other natural sources.

## 2. Polysaccharides

**Figure 1:**
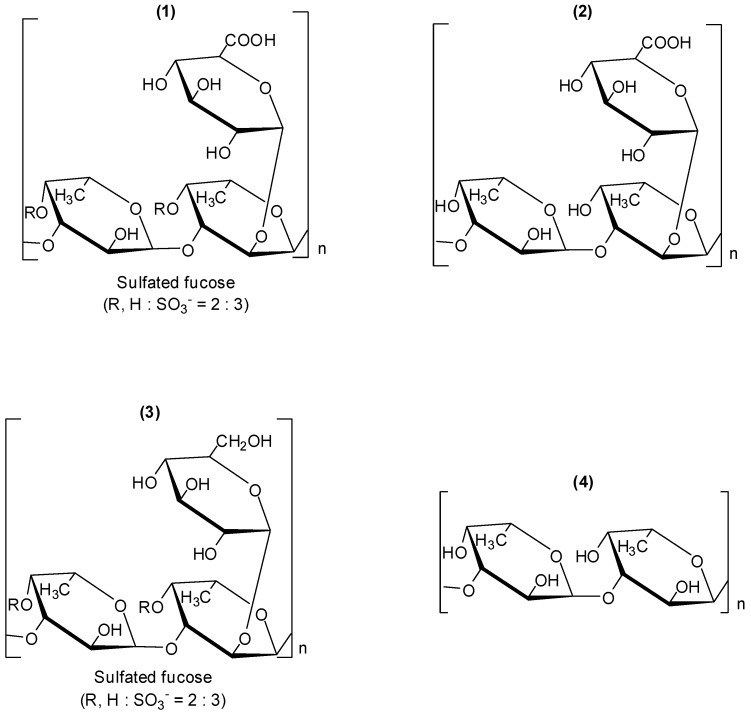
Fucoidan (**1**) and derivatives **2**–**4**.

Fucoidans are a group of polysaccharides which contain considerable percentages of L-fucose and sulfate ester groups. These compounds are mainly derived from brown seaweed, and several bioactivities have been described for them, including antiviral ones [28,29,30]. Fucoidan (**1**, Figure 1), is a polysaccharide isolated from the marine alga brown seaweed *Cladosiphon okamuranus*. Its structure is composed of repeating units of sulphated fucose and glucuronic acid residues. The investigation conducted with *Cladosiphon* fucoidan (**1**) demonstrated that this polysaccharide inhibits dengue virus type 2 (DENV-2) infection [31]. The biological assays to evaluate antiviral activity were conducted *in vitro* by focus-forming assay using BHK-21 cells. Compound **1** inhibited virus infection in a concentration-dependent matter. When the virus was treated with 10µg/mL of fucoidan (**1**), infectivity by dengue virus serotype 2 was reduced by 80% compared with that in untreated cells and the determined IC_50_ corresponded to 4.7 µg/mL. Dengue virus serotypes 3 and 4 were moderately susceptible to **1**. For serotype 1, fucoidan (**1**) did not present an effect on the infection. Fucoidan derivatives (**2**–**4**) (Figure 1) were also examined for their effects on infection of BHK-21 cells by dengue virus serotype 2. Polysaccharide **2** is a derivative obtained by removal of the sulfated groups in fucoidan (**1**); derivative **3** was prepared by reduction of fucoidan (**1**). Fucan (**4**) is a fucose polymer.

The desulfation of fucoidan (**1**) led to derivatives **2** and **4** which showed remarkable suppression of inhibitory activity. This finding is in agreement with previous investigations which demonstrated that sulfation is required for the antiviral activity of glycosaminoglycans [32]. Even though compound **3** is a sulfated derivative, the reduction of carboxylic acid functionality in fucoidan (**1**) to the corresponding alcohol also resulted in a decrease of the capability of **3** in preventing serotype 2 virus infection. Therefore, it was concluded that the glucuronic acid residue as well as the sulphate groups are fundamental for the inhibitory activity of fucoidan (**1**) against DENV-2 [31]. It was also reported that glucuronic acid and sulfated fucose residues of the *Cladosiphon* fucoidan appear to critically affect the interaction of DENV-2 with cellular receptors, but the precise molecular mechanism of the inhibitory effects of this compound has not been elucidated.

**Figure 2:**
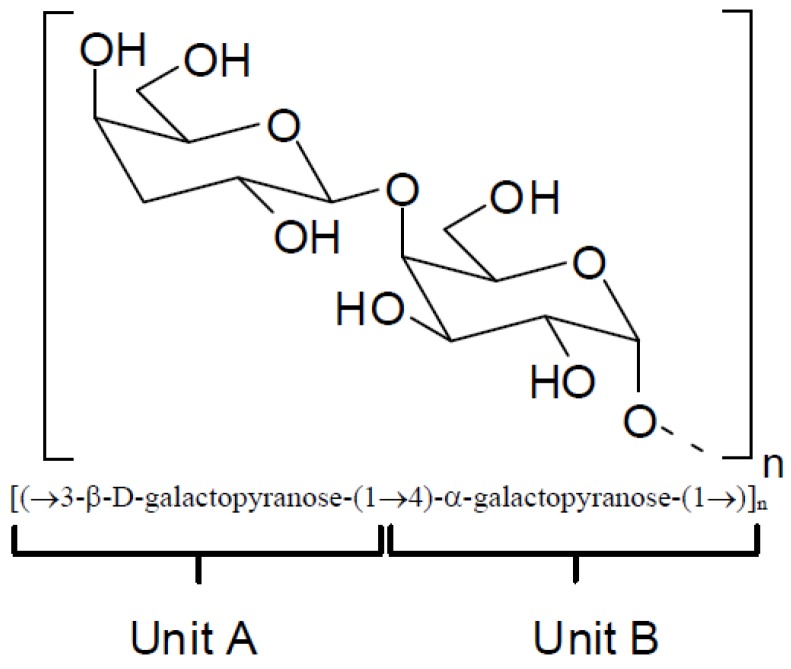
Repeating basic structure of galactans.

Several polysaccharides known as galactans have been isolated from red seaweeds [33,34,35,36]. The structures of these compounds correspond to a linear chain of β-d-galactopyranose residues linked by positions 1 and 3 (Figure 2, unit A) and residues of α-galactopyranose linked by positions 1 and 4 (Figure 2, unit B) resulting in an arrangement in which units A and B are alternating. Natural chemical modifications in these structures include the presence of sulfate esters groups, pyruvate acetal and/or methyl ethers. In addition, unit B can exist in the 3,6-anhydro-α-galactopyranose form.

**Figure 3:**
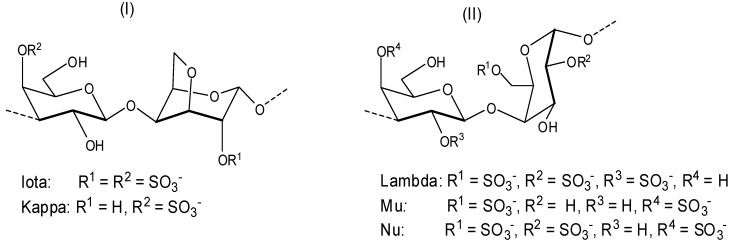
Repeating structures of carrageenans.

**Figure 4:**
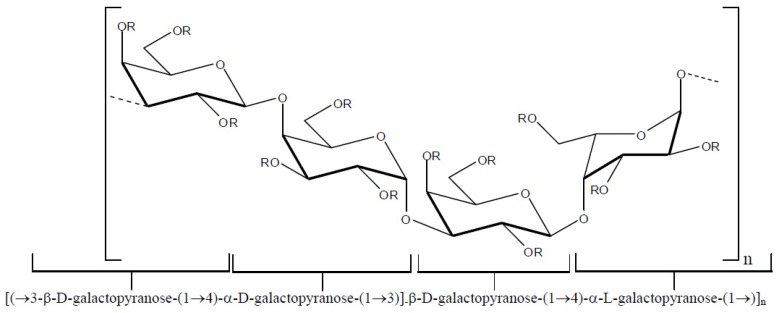
Basic repeating unit of dl-hybrid galactans.

Depending on the stereochemistry of unit B (Figure 2), the galactans can be classified as follows: (i) carrageenans—these correspond to sulfated polysaccharides with 4-linked α-galactose residues of the d-series or their 3,6-anhydro derivatives (Figure 3). Carrageenans are typically classified according to their structural features, including their sulfation patterns and the presence or absence of 3,6-anhydro-α-galactopyranose on d-units. There are at least 15 different carrageenan structures, the most relevant being *iota*, *kappa* [Figure 3, (I)], *lambda*, *mu*, and *nu* [Figure 3, (II)] [37]. It is important to mention that natural carrageenans typically occur as mixtures of different hybrids. Moreover, methyl or pyruvic acid acetal moieties and the presence of small amounts of other sugars can add to the structures of these polysaccharides [37].

(ii) agarans—this group of galactans differs from carrageenans in terms of the stereochemistry of unit B. For the agarans, the 4-linked α-galactose residues unit b correspond to the l-series.

(iii) dl-hybrids—these galactans are characterized by the presence of 4-linked α-galactose residues of the d and l series in the same molecule. Figure 4 depicts the basic repeating structure of dl-hybrid galactans.

Commercially available *iota*, *kappa* and *lambda* carrageenans (Figure 3) were evaluated against DENV 1–4 serotypes [38]. The assays were conducted *in vitro*, using Vero and HepG2 cells and the activity of carrageenans was initially tested by plaque reduction assays, necessarily performed at a low multiplicity of infection (m.o.i.), but then confirmed by inhibition of virus yield and antigen expression assays, accomplished at high m.o.i. The polysaccharides were more effective on DENV-2 and DENV-3 serotypes. It was also determined that the carrageenans *lambda* and *iota* are potent inhibitors of DENV-2 and DENV-3 multiplication in Vero and HepG2 cells with EC_50_ (effective concentration 50%) ranging from 0.14 to 4.1 µg/mL (Table 1). The results showed that the lack of dependence of the antiviral potency of carrageenans on the infecting virus inoculum was even more evident when the assays were performed simultaneously at a wide range of multiplicities. This important property represents a clear advantage for those compounds able to block infection even in the presence of high initial virus doses.

**Table 1:** EC_50_ values for inhibition of DEN-2 and DENV-3 multiplication in Vero and HepG2 cells.

Polysaccharide	EC_50_ (µg/mL)	
	Vero Cells	HepG2 cells
DENV-2		
*iota*-carrageenan	0.4 ± 0.1	0.14 ± 0.01
*lambda*-carrageenan	0.22 ± 0.02	0.17 ± 0.01
DENV-3		
*iota* -carrageenan	1.1 ± 0.1	0.63 ± 0.01
*lambda* -carrageenan	0.6 ± 0.1	0.63 ± 0.01

This study was able to demonstrate that a heparin sulfate (HS) imitative compound *lambda* had the ability to interfere with DENV-2 replication when added after virus adsorption, and even under these conditions, the antiviral potential of *lambda*-carrageenan was higher than its ability to affect virus adsorption. The mechanism of the inhibitory multiplication effect of the *iota* carrageenan was not described.

**Figure 5:**
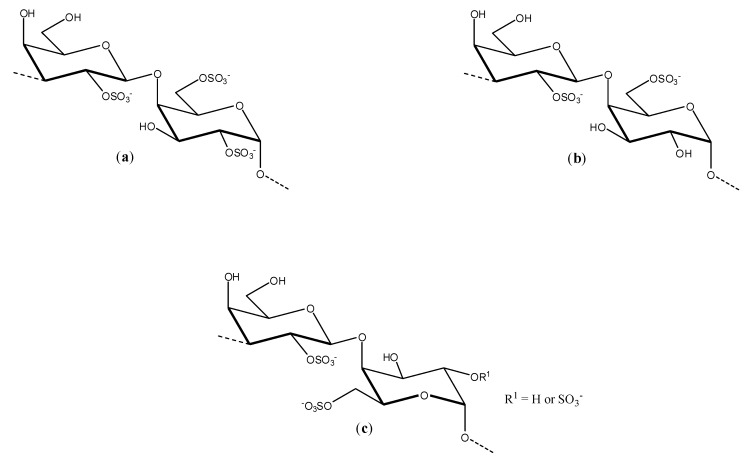
Disaccharides units present in the structure of *C2S-3*.

Talarico and co-workers investigated the dengue antiviral activity of two sulfated polysaccharides obtained from the read seaweeds *Gymnogongrus griffithsiae* and *Cryptonemia crenulata* [39]. The *G3d* compound obtained from *G. griffithsiae* is a carrageenan composed of *kappa*/*iota*/*nu* repeating units (see Figure 3). The DL-galactan hybrid *C2S-3* obtained from *C. crenulata* is made of the units disaccharide units (**a**), (**b**), and (**c**) shown in Figure 5.

The antiviral activity evaluation of *G3d* and *C2S-3* was conducted *in vitro* and it was carried out by two methods: plaque reduction and virus yield inhibition assays. It was found that the dengue antiviral activity of sulfated *G3d* and *C2S-3* polysaccharides depends on both virus serotype and the host cell. Both compounds were capable of inhibiting DENV-2 in Vero cells with IC_50_ values for the inhibitory activity close to 1 μg/mL and selectivity indexes superior to 1,000. The compounds were completely ineffective against DENV-1 and their inhibitory activity on DENV-3 (IC_50_ ranging from 13.9–14.2 μg/mL) and DENV-4 (IC_50_ ranging from 29.3 to > 50 μg/mL) were significantly lower. The inhibitory effects of *G3d* and *C2S-3* against DENV-2 and DENV-3 were also evaluated with respect to human HepG2 and foreskin PH cells as well as mosquito C6/36 HT cells. While the polysaccharides were totally ineffective against mosquito cells, the effectiveness of the substances with respect HepG2 and PH cells were similar to that observed with Vero cells. From the mechanism of action standpoint, it was determined that *G3d* and *C2S-3* present inhibitory effects on DENV-2 serotype only when they are added together with the virus or early after infection. Therefore, the processes of virus adsorption and internalization are the main targets of these compounds. The results suggest that these compounds act on virus binding. These polysaccharides may be useful tools to elucidate the mechanisms of binding and internalization of DENV serotypes to vertebrate and invertebrate cells, and also to establish structure-activity relationships [39].

Another study involving sulfated polysaccharides showed that the extracts and carrageenans derived from *Meristiella gelidium* were more effective inhibitors of DENV-2 compared to those derived from *G. griffithsiae*. The antiviral property evaluation was performed *in vitro* using Vero cells and virus plaque reduction assay. In this investigation, no description on the mechanism of action for DENV inhibition was reported [40].

**Figure 6:**
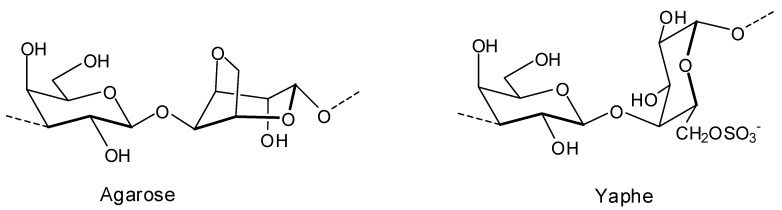
Agarose and Yaphe repeating units found in dl-galactan hybrids isolated from the red seaweed *G. torulosus*.

A series of DL-galactan hybrids isolated from the red seaweed *Gymnogongrus torulosus* was assessed *in vitro* against DENV-2 serotype by virus reduction assay in Vero cells [41]. The repeating disaccharide units and the percentage of them found in the composition of the polysaccharides are presented in Table 2. Structures of agarose and Yaphe repeating units are shown in Figure 6.

**Table 2:** .dl-galactan hybrids from *G. torulosus* and their IC_50_ values when the galactans were evaluated against DENV-2 virus serotype.

Polyssacharide	MW(kDa) ^a^	Galactan Structure				IC_50_(μg/mL) ^c^
		Kappa/iota ^b^	mu/nu ^b^	Agarose ^b^	Yaphe ^b^	
C1	44	57	10	19	12	1.1 ± 0.2
C2	56	1845	18	22	12	0.7 ± 0.2
C3	77	28	21	31	14	0.34 ± 0.06
C4	18	54	6	7	17	0.19 ± 0.03
F1	56	76	-	19	12	0.5 ± 0.2
F2	77	73	12	12	-	0.8 ± 0.1
F3	18	34	-	12	51	0.9 ± 0.2
F3T2	35	62	-	32	20	0.25 ± 0.09
F3T4	45	38	12	40	9	0.34 ± 0.02
F3T6	22	17	8	8	59	1.7 ± 0.1

^a^ Average number molecular weight. ^b^ Approximately percentage. ^c^ Inhibitory concentration required to reduce plaque number in Vero cells by 50%. Values are the mean of two determinations ± standard deviation

As can be seen in Table 2, the evaluated polysaccharides were very active against DENV-2 serotype with IC_50_ ranging from 0.19–1.7 μg/mL. In addition to their inhibitory activity, the compounds did not present cytotoxic effects on stationary or on actively dividing cells, and they presented anticoagulant properties. It is suggested that the mechanism of action of these compounds corresponds to interference in the binding of the surface glycoprotein with the cell receptor [41].

One aspect deserves comment at this point. Liang and co-workers prepared sulfated agarose, sulfated *kappa*-carrageenan, desulfated *kappa*-carrageenan, and *kappa*-carrageenan oligosaccharides to investigate their anticoagulant and cytotoxic activities. They found that anticoagulant activity and effects on cell proliferation are both dependent on the substitution position rather than the degree of sulfate group substitution. Moreover, these activities are dependent on the secondary structures of polysaccharides. The investigation led to the conclusion that carrageenan and agarose can be considered for biomedical applications after careful tailoring of sulfate groups [42].

**Figure 7:**
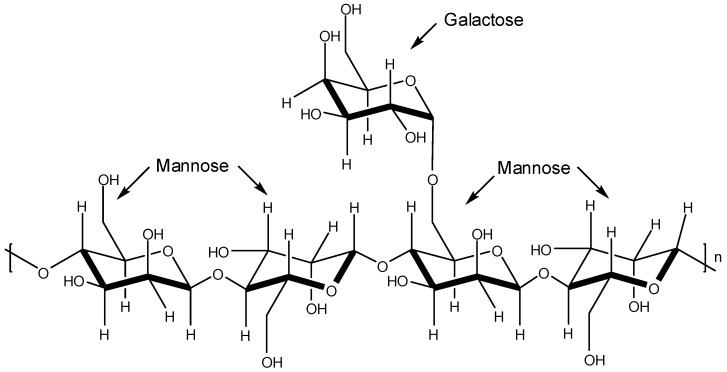
Basic structure of galactomannans.

The plant storage polysaccharides known as galactomannans are characterized by a main chain of β-d-mannopyranosyl residues linked by positions 1 and 4 with single unit α-d-galactopyranosyl side-chain-residues (Figure 7). The mannose/galactose ratio depends on either the plant source or the extraction method used [43,44].

Ono and co-workers isolated two sulfated galactomannans [45]. One was isolated from the seeds of *Mimosa scabrella* presenting a mannose/galactose ratio of 1.1 and the other was obtained from the seeds of *Leucaena leucocephala* (mannose/galactose ratio of 1.4). These sulfated polysaccharides named by the authors as BRS (from *M. scabrella*) and LLS (from *L. leucocephala*) were submitted to *in vitro* and *in vivo* assays to assess their effects on DENV-1 (Hawaii strain) virus. The *in vivo* assay was carried out with female mice to determine antiviral activity. The *in vitro* evaluation was conducted with C6/36 cells by virus plaque reduction. Death of DENV-1-infected mice was not noticed. For the *in vitro* assay, it was found that the concentrations that produced a 100-fold decrease in virus titer were 347 mg/L and 37 mg/L for BRS and LLS, respectively [45].

## 3. Flavonoids

**Figure 8:**
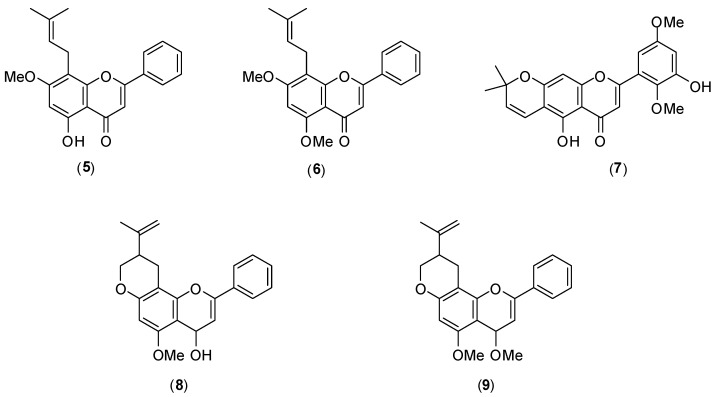
Structures of flanovoids from Mexican *Tephrosia* species

Several flavonoids have been screened for dengue antiviral activity. Figure 8 shows the structures of members of this class of compounds **5**–**9**, isolated from Mexican *Tephrosia* species. Their antiviral activity was evaluated *in vitro* by the plaque assay using LLC-MK_2_ cells and DENV-2 serotype. Of the five flavonoids tested, only glabranine (**5**) and 7-*O*-methylglabranine (**6**) showed significant inhibitory activity (they presented 70% virus infection inhibition at 25 µmol/L). No IC_50_ values were reported. The other flavonoids **7**–**9** had no antiviral effect. Considering the structures of compounds **5**–**9**, it is evident that a relationship exists between the structure and antiviral activity of the investigated flavonoids since glabranine (**5**) and 7-*O*-methylglabranine (**6**), which both contain a prenyl side-chain at C-8, were active as replication inhibitors [46].

**Figure 9:**
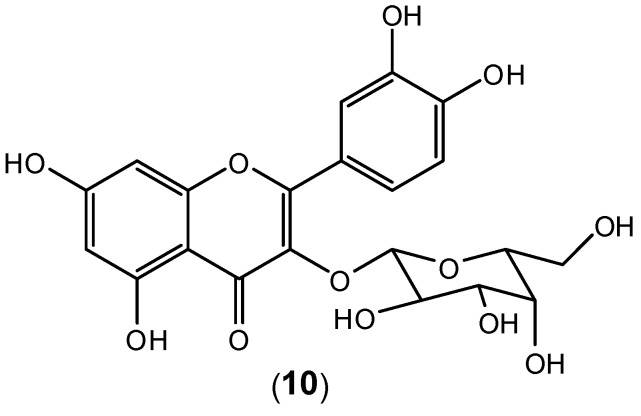
Structure of hyperoside (**10**).

The aqueous extract of *Houttuynia cordata*, a popular side dish vegetable consumed in Northern and Eastern regions of Thailand, was tested against DENV-2 [47]. The assays to evaluate antiviral aqueous activity were performed *in vitro* in three different modes: (i) protective, (ii) treatment, and (iii) direct blocking in HepG2 and LLC-MK_2_ cells. For (i) and (ii) the experiments were conducted using plaque titration assay, and for (iii) it was performed using plaque reduction assay. Considering HepG2 cells, *H. cordata* aqueous extract displayed an inhibitory effect to DENV-2 RNA production in all experimental modes. The experiments were conducted at 10 μg/mL and 100 μg/mL. The higher concentration was effective to: (a) protect HepG2 cells from DENV-2 infection (protective mode); (b) decrease the intracellular viral RNA synthesis (treatment mode); (c) to inactivate the virus (direct blocking). The greatest inhibitory effect was observed in the protective mode. For LLC-MK_2_ cells, the experiments were conducted within 10–40 μg/mL concentration range, and the aqueous extract also exhibited a protective effect on virion release. High Performance Liquid Chromatography (HPLC) analysis identified the flavonoid hyperoside (**10**) as the major component of the extract (Figure 9). It is very likely that the observed dengue antiviral activity is associated with the presence of this flavonoid as the major component in the aqueous extract. The extract also protects the cells from viral entry and inhibits virus activities after adsorption. In the treatment mode, hyperoside (**10**) in the extract probably inhibited intracellular RNA synthesis by interacting with enzymes or proteins in the viral replication complex [47]. Apparently pure hyperoside-even though quite readily available from many plants- has not been tested as an isolated compound.

**Figure 10:**
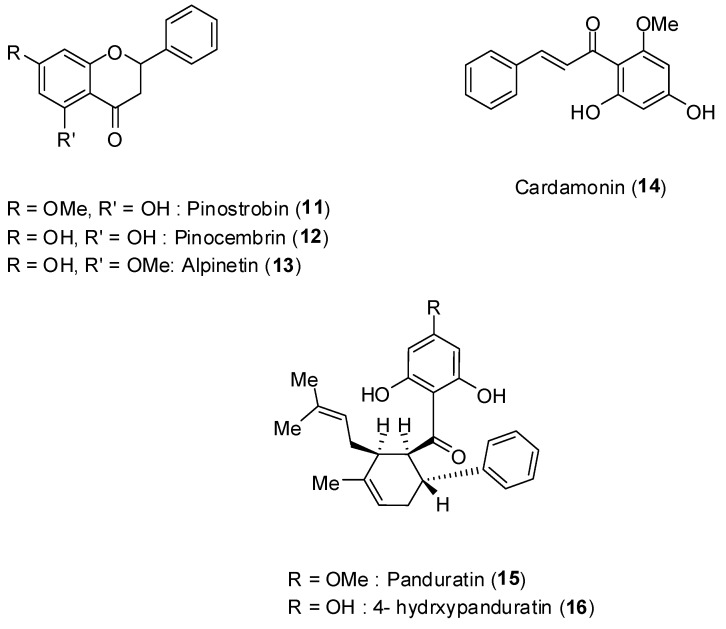
Structures of compounds **11**–**16**.

*Boesenbergia rotunda* (L.) is a common spice belonging to the ginger family (Zingiberaceae). Figure 10 depicts the structures of the flavonoids pinostrobin (**11**), pinocembrin (**12**), alpinetin (**13**), the phenylpropanoid cardamonin (**14**), and the cyclohexenylchalcone derivatives pandurantin A (**15**) and 4-hydroxypanduratin A (**16**), all of them isolated from the aforementioned vegetal species [48].

These natural products were screened against DENV-2 virus NS2B-NS3 protease by enzyme assay. The assay was performed with the purified protease [48]. Increased concentration of the compounds led to increase inhibition of enzyme activity. Among the compounds, pinocembrin (**12**) was the least active inhibiting by about 60% at 400 ppm concentration. The most active substances corresponded to panduratin A (**15**) and 4-hydroxypanduratin B (**16**), and their inhibitory protease activities are presented in Table 3.

**Table 3:** Percentage inhibition of DENV-2 NS2B-NS3 virus protease cleavage ^a^ of compounds and **15** and **16**.

Compound	Percentage Inhibition of DENV-2 NS2B-NS3 Protease; Concentration Used (ppm)					
	40	80	120	160	240	400
15	27.1 ± 4.8	66.7 ± 0.1	87.7 ± 0.6	93.7 ± 0.5	92.12 ± 1.2	99.8 ± 1.1
16	52.0 ± 1.1	78.1 ± 0.1	87.6 ± 0.4	96.0 ± 0.5	97.3 ± 0.3	99.8 ± 0.3

^a^ The substrate cleaved by NS3 protease used in the experiment corresponded to the peptide Boc-Gly-Arg-Arg-MCA. ± corresponds to standard deviation.

Interestingly, although the compounds **12** and **14** had low inhibitory activity on NS2B-NS3 protease, some synergistic effect was noticed when these compounds were mixed (Table 4).

**Table 4:** Percentage inhibition of DENV-2 NS2B-NS3 virus protease cleavage ^a^ of compounds and **12** and **14**.

Compound	Percentage Inhibition of DENV-2 NS2B-NS3 Protease; Concentration Used (ppm)		
	120	240	400
12	30.1 ± 0.5	47.3 ± 0.5	56.1 ± 0.4
14	39.4 ± 0.6	50.1 ± 0.4	71.3 ± 0.3
12 + 14	52.6 ±0.4	63.5 ± 0.5	81.8 ± 0.3

^a^ The substrate cleaved by NS2B-NS3 protease protease used in the experiment corresponded to the peptide Boc-Gly-Arg-Arg-MCA. ± corresponds to standard deviation.

For the most active compounds **15** and **16**, it was determined that they present competitive inhibitory activity on NS2B-NS3 displaying inhibitory constants (*K_i_*) of 21 and 25 μmol/L, respectively.

**Figure 11:**
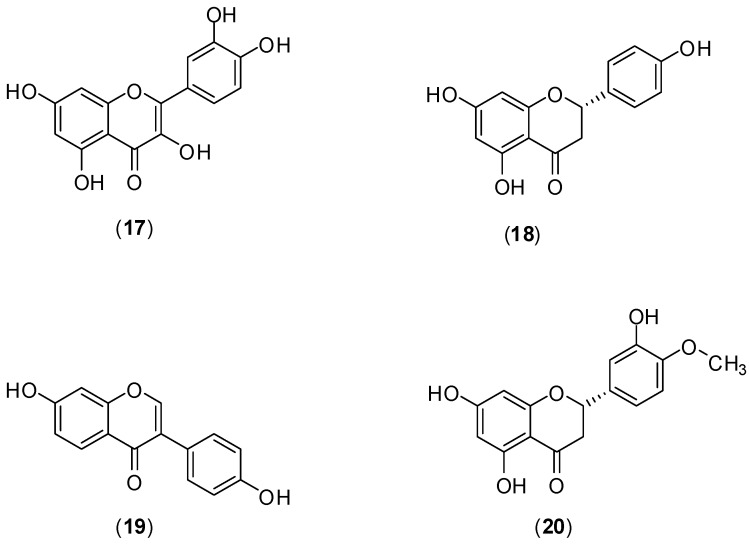
Structures of compounds **17**–**20**.

The flavonoids quercetin (**17**), naringenin (**18**) daidzein (**19**), and hesperetin (**20**) (Figure 11) were evaluated against DENV-2 serotype by Zandi and co-workers. The assessment of antiviral activity was conducted *in vitro* utilizing Vero cells. DENV replication was measured by Foci Forming Unit Reduction Assay (FFURA) and quantitative real time polymerase chain amplification (qRT-PCR). [49]. Of these compounds, only quercetin (**17**) presented significant inhibitory activity (IC_50_ was 35.7 μg/mL) against DENV-2 infection in Vero cells. The selective index for quercetin when the infected cells were treated or when uninfected cells were treated continuously 5h before infection until 4 days post infection were 7.07 and 8.74, respectively. The mechanism by which quercetin exerts its antiviral effect remains unknown. However, it is believed that DENV antiviral activity of **17** could be similar to that presented by others flavonoids that act against cellular RNA polymerases and formation of the complex with RNA [49].

**Figure 12:**
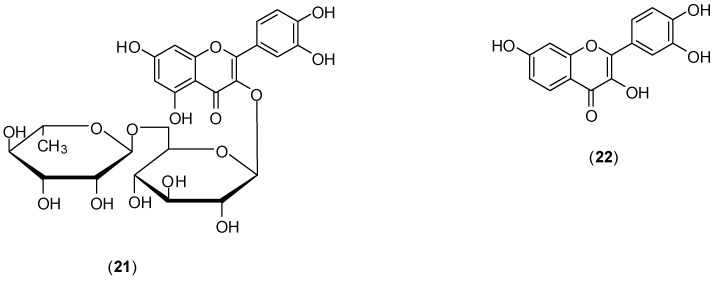
Structures of rutin (**21**) and fisetin (**22**).

The study of Zandi and co-workers evaluated the effects of naringenin (**18**) (Figure 11), rutin (**21**), and fisetin (**22**) (Figure 12) against DENV-2 (NGC strain). Antiviral effects of each compound at the different stages of DENV-2 infection were examined *in vitro* using FFURA and qRT-PCR. The authors found that among the flavonoids studied only **22** presented significant *in vitro* activity on dengue virus replication activity [50]. Compound **22** did inhibit virus replication (IC_50_ of 55 µg/mL and selectivity index of 4.49) after virus adsorption on Vero cells. When Vero cells were continuously treated for 5 h before virus infection and continuously up to 4 days post-infection, IC_50_ corresponded to 43.12 µg/mL and selective index of 5.72. The authors report that no virucidal or prophylactic activity was noticed for fisetin (**22**). Even though compounds **18** and **21** did not inhibit DENV-2 replication, flavonoid **18** displayed virucidal activity (IC_50_ of 52.64 µg/mL), albeit with a low selectivity index (<1). The mechanism of how fisetin affects DENV virus replication is unclear. For the authors, it is not likely to act directly on the virus, because it does not affect DENV-2 binding to cells. They believe that fisetin (**22**) could affect DENV genome copy number by interference in DENV-2 replication (by binding directly to virus RNA or forming a flavonoid-RNA complex) or by affecting the RNA polymerases resulting in inhibition of virus replication [50].

A biological screening conducted on 1,350 ethyl acetate extracts prepared from various parts of approximately 650 New Caledonian plants was carried out targeting the DENV-2 NS5 polymerase. Enzyme assays were performed. DENV-2 NS5 polymerase genes were tagged by six N-terminal histidine residues and expressed from the pQE30 vector (Qiagen, Venlo, Limburg, Netherlands) in *E. coli* Rosetta pLacI cells (Novagen, Darmstadt, Germany). The enzymes were produced and purified by Heparin-Sepharose Chromatography. Alternatively, gel filtration was used as a second purification step.

**Figure 13:**
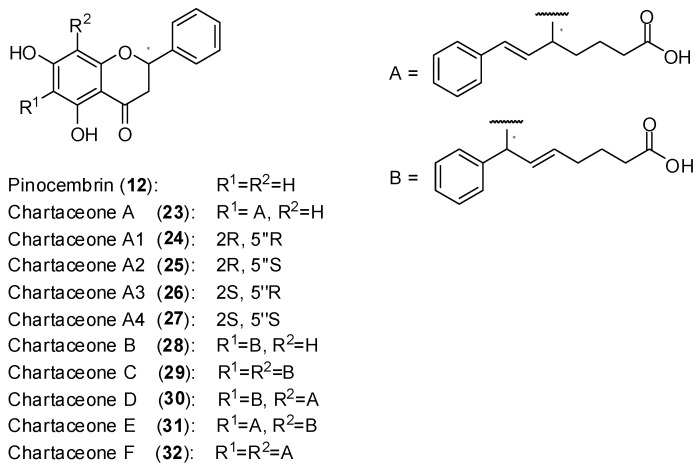
Structures of pinocembrin and chartaceones.

**Figure 14:**
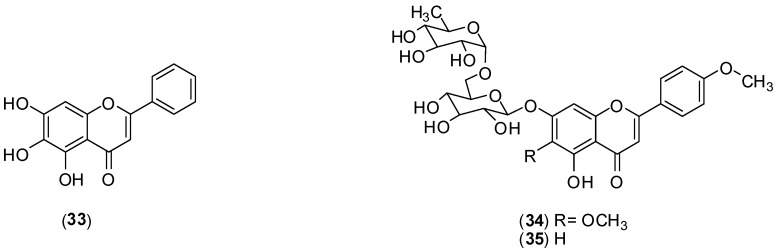
Structures of compounds **33**–**35**.

Polymerase activity was assayed by monitoring the incorporation of radiolabeled guanosine into a homopolymeric cytosine RNA template. Among several active extracts, the very active one from the bark of *Cryptocarya chartacea* Kostern, a species belonging to the Lauraceae family [51] was selected. From the selected extract, it was isolated the non-alkylated flavonoid pinocembrin (**12**) as well as series of new mono and dialkylated ones named chartaceones A-F (**23**–**32**, Figure 13). The screening of compounds **23**–**32** against DENV-2 NS5 polymerase showed that the chartaceones C-F (compounds **29**–**32**) were the most active in inhibiting polymerase activity (IC_50_ ranging from 1.8 to 4.2 µmol/L) while the other chartaceones were less effective. On the contrary, compound **12** was completely inactive. These findings suggest that the presence of alkylated chains in the structures of chartaceones C-F (**29**–**32**) play important role in terms of inhibitory activity on DENV-2 NS5 polymerase. The compounds **12**, **23**–**33** were also screened against bovine diarrhea virus (BVDV) NS5 polymerase and no activity was observed. Therefore, it seems that these natural substances present some selectivity towards DENV2-NS5 polymerase. Considering that the activity of compounds **29**–**32** against DENV-2 NS5 polymerase was similar, it was concluded that side chains A and B (Figure 13) play an equivalent role in terms of biological activity [51]. Figure 14 shows the structures of the last two compounds discussed in this section.

*Scutellaria baicalensis* is a tradicional Chinese medicinal herb belonging to the Lamiaceae family. From the roots of this species, baicalein (**33**) is usually extracted [52]. The authors conducted *in vitro* assay using Vero cells an FFURA to assess antiviral activity against DENV-2. This flavonoid inhibited DENV-2 serotype replication in Vero cells displaying an IC_50_ of 6.46 µg/mL and a selectivity index of 17.8 when it was added after adsorption to the cells. The IC_50_ against DENV-2 is 5.39 µg/mL and the selectivity index increased to 21.3 when Vero cells were treated before virus infection and continuously up to 4 days post-infection. Substance **33** displayed direct virucidal (IC_50_ of 1.55 µg/mL) as well as anti-adsorption (IC_50_ of 7.14 µg/mL) effects against DENV-2. The results suggest that a possible mechanism for the extracellular and intracellular activities of baicalein (33) against DENV-2 could be attributed to its ability to bind and/or to inactivate important structural and/or non-structural protein(s) of DENV-2 [52].

A phytochemical investigation of the ethanol extracts from *Distictella elongate* (Vahl) Urb led to isolation of petcolinarin (**34**) from the leaf extract and a mixture of **34** and acacetin-7-*O*-rutinoside (**35**) from fruit extract. *In vitro* MTT colorimetric assays, using Vero and LLCMK2 cells, were conducted to assess antiviral activity against DENV-2. The mixture of **34** and **35** presented better anti-DENV-2 activity (EC_50_ of 11.1 ± 1.6 µg/mL and selectivity index > 45) than pure petcolinarin (**34**) (EC_50_ of 86.4 ± 3.8 µg/mL and selectivity index of 4.6). The mechanism of inhibition of the compounds is unclear, but it is suggested that it may correspond to one of the putative mechanisms already described for flavonoids [53].

## 4. Alkaloids and Related Compounds

**Figure 15:**
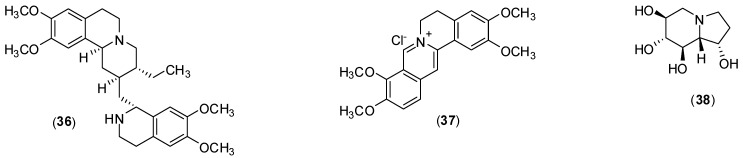
Structures of alkaloids **36**–**38**.

Emetine (**36**, Figure 15) is a compound belonging to the ipecacuanha alkaloids. Its dihydrochloride was identified by Low and co-workers as a compound displaying potent DENV antiviral activity at a very low concentration of 0.5 µmol/L (277 ng/mL) [54]. The investigators conducted *in vitro* experiments using Huh-7 and BHK21 cells via viral plaque assay associated with immunofluorescence assay and qRT-PCR. A series of experiments led to the conclusion that emetine dihydrochloride acts inhibiting DENV infection at the early stages of viral replication life cycle, either affecting the viral RNA synthesis pathway or viral protein translation pathway.

*Coptis chinensis Franch* is a medicinal plant used in China to treat bacterial, inflammatory, fungal and other diseases, presenting no significant side effects or toxicity to humans at clinical doses [55]. This vegetal species presents a high concentration of palmatine (**37**, Figure 15) which was screened *in vitro* for its antivirus activity against DENV-2 using Vero cells via viral titer reduction assays. Vero cells were infected with DENV-2 and the EC_50_ was estimated to be 26.4 µmol/L and the selectivity index to be 39. In the same work, the authors demonstrated in an enzyme assay that palmatine (**37**) could inhibit the NS2B-NS3 protease of West Nile Virus (WNV). The mechanism by which alkaloid **37** inhibits the virus is not clear yet; the authors of this investigation plan to clarify the mechanism of action mainly based on a viral reverse genetics system, virus-encoded proteases and selection and characterization of palmatine-resistant viruses [55].

The water soluble castanospermine (**38**), Figure 15, is derived from *Castanospermum australe* (black bean or Moreton Bay chestnut tree). An *in vitro* and *in vivo* experiments were conducted to ascertain whether this alkaloid can inhibit all dengue virus serotypes. The *in vitro* experiment used to investigate antiviral activity used BHK-21 cells in a plaque reduction assay and was verified with western blotting, ELISA and fluorogenic RT-PCR. *In vivo* experiment was also conducted with A/J mice (28 to 31 days old). Alkaloid **38** inhibits all dengue virus serotype infections *in vitro* and dengue virus serotype 2 *in vivo*. It was found that inhibition occurs at the level of secretion and infectivity of viral particles. Additionally, castanospermine (**38**) prevented mortality in a mouse model of dengue virus infection, with doses of 10, 50, and 250 mg/kg of body weight per day being highly effective at promoting survival [56].

**Figure 16:**
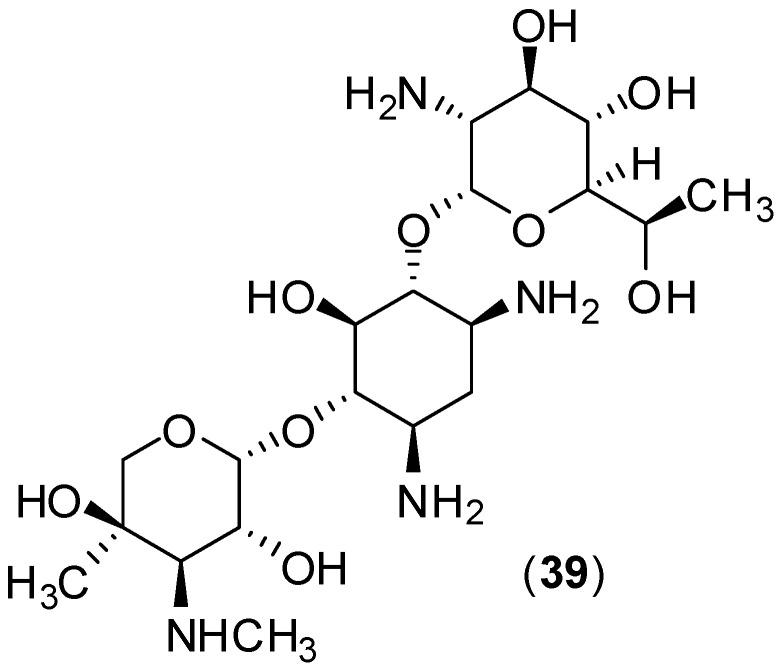
Structure of the aminoglycoside geneticin (**39**).

The aminoglycoside geneticin (**39**, Figure 16) presents antiviral activity against bovine viral diarrhea virus (BVDV). Taking into consideration that dengue virus, yellow fever virus and BVDV are in the *Flaviviradae* family, Zhang and co-workers undertook the task of screening **39** against dengue virus.

The antiviral activity was assessed *in vitro* with BHK cells by viral plaque reduction assay with western blotting and qRT-PCR. The results demonstrated that compound **39** inhibits DENV-2 proliferation by: (i) protecting BHK cells against the cytopathic effect of DENV-2 (EC_50_ of 3 ± 0.1 µg/mL for this activity); (ii) reducing the viral yield (EC_50_ of 2 ± 0.1 µg/mL and EC_90_ of 20 ± 2 µg/mL); (iii) inhibiting DENV-2 plaque formation in both the number and the size of the plaques (EC_90_ of 25 µg/mL); (iv) blocking DENV-2 RNA and protein synthesis. It was also found that the selectivity index of **40** was equal to 66 [57]. While compound **40** was active against DENV-2, no inhibition was observed on yellow fever virus in the screening with BHK cells. The molecular mechanism of antiviral activity of geneticin (**40**) remains unclear. The results suggest that the geneticin-mediated antiviral mechanism is cell type-independent [57].

## 5. Terpenoids

**Figure 17:**
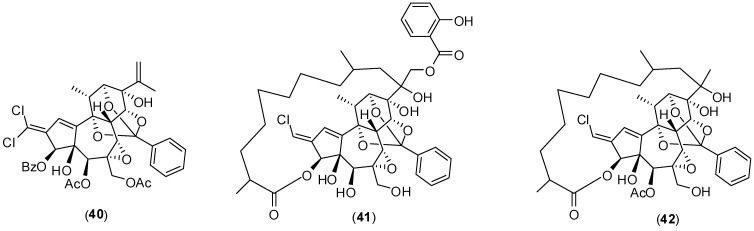
Structures of terpenes **40**, **41** and **42**.

The bark and the wood of *Trigonostemon cherrieri*, a rare plant of New Caledonia, were investigated for their chemical composition resulting in the isolation and characterization of several oxygenated terpenes , among them compounds **40**–**42** (Figure 17).

The compounds were evaluated for the ability to interfere with NS5 DENV polymerase by an enzyme assay with purified enzyme. All of them indeed presented inhibitory effects on enzyme activity with the IC_50_ of 12.7 ± 0.2, 3.1 ± 0.2 and 16.0 ± 1.3 µmol/L for compounds **40**, **41**, and **42**, respectively. There is no report on the mechanism of inhibition of the compounds [58].

**Figure 18:**
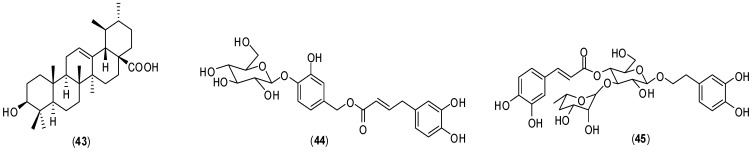
Structure of terpenoid **43** and phenolic compounds **44** and **45**.

A bioguided investigation aimed to obtain antiviral chemical constituents from an ethanol extract of leaves from *Arrabidaea pulchra* resulted in the isolation of triterpene compound **43** along with phenolic derivatives **44** and **45** (Figure 18).

The isolated compounds displayed activity against DENV-2. Cytotoxity was determined *in vitro* against LLCMK_2_ and Vero cells by MTT colorimetric assay. The determined EC_50_ and the selectivity indexes for the compounds were as follows: **43** (EC_50_ = 3.2 ± 0.6 µg/mL; selectivity index = 3.1); **44** (EC_50_ = 2.8 ± 0.4 µg/mL; selectivity index = 20.0); **45** (EC_50_ = 3.4 ± 0.4 µg/mL; selectivity index = 3.8). The same assay was conducted with Human Herpesvirus-1 (HSV-1), Vaccinia Virus Western Reserve (VACV-WR) and Murine Encephalomyocarditis virus (EMCV). The inhibition of HSV-1 and VACV-WR was lower than that of DENV-2, and no inhibition was observed for EMCV. Further investigations are needed to understand the mechanisms of the antiviral activity, especially for compound **45** which displayed the lowest toxicity in LLCMK2 cells and was active only against DENV-2. Further assays are also needed to investigate virucidal activity and targets in the viral replication cycle [59].

## 6. Polycyclic Quinones

**Figure 19:**
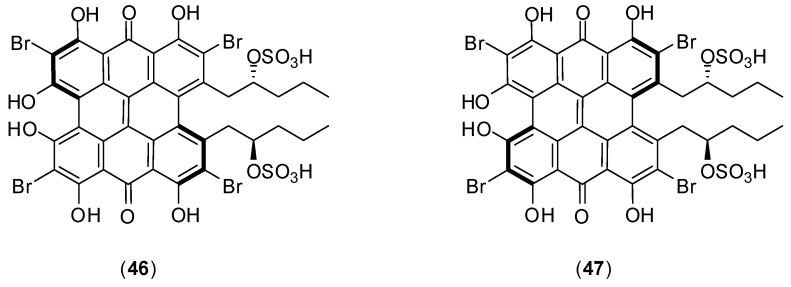
Structures of polycyclic quinones **46** and **47**.

The marine environment has been explored in the interests of identification of new leads for pharmaceutical purposes including antivirals [60]. The polycyclic gymnochrome D (**46**) and isogymnochrome D (**47**) (Figure 19**)** were isolated from the living fossil crinoids *Gymnocrinus richer*i [61]. The assessment of their effect on DENV-1 (strain Hawai/1944) virus was conducted *in vitro* using porcine PS cells in a plaque reduction assay. The results showed that they display antiviral activity. This activity was determined by the reduction of foci (RF) formed by DENV-1 compared with controls. Thus, for compound **46** and **47**, the determined RF_50_ was smaller than 1 µg/mL. The mechanism by which the compounds could inhibit the virus was not reported [61].

**Figure 20:**
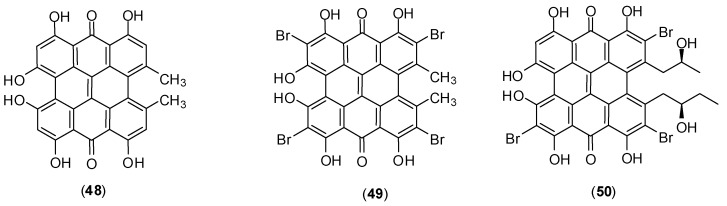
Structures of polycyclic quinones **48**, **49** and **50**.

The polycyclic quinones hypericin (**48**), tetrabromohypericin (**49**) and gymnochrome B (**50**) (Figure 20) were also examined for both their antidengue virus activities and their photoactivities. In this investigation researchers conducted an *in vitro* experiment with PS cells by plaque reduction assay against dengue virus serotypes 2 and 4. It was determined that all of these quinones presented virucidal an antiviral activities which are enhanced by light. The virucidal activity presented the following increasing order of potency: tetrabromohypericin (**49**) (ED_50_ = 2.8 nmol/mL); hypericin (**48**) (ED_50_ = 1.8 nmol/mL); gymnochrome B (**50**) (ED_50_ = 0.042 nmol/mL). A similar trend was found for the antiviral effect: tetrabromohypericin (**49**) (ED_50_ = 3.7 nmol/L); hypericin (**48**) (ED_50_ = 0.6 nmol/mL); gymnochrome B (**50**) (ED_50_ = 0.029 nmol/L). ED stands for effective dose. The tested doses ranged from 50 to 0.001 µg/mL. Considering that the most active compound was gymnochrome B (**50**), it is apparent that the presence of the side chain in the structure of compound **50** seems to be beneficial for both virucidal and antiviral activities. [62].

## 7. Phenolics

**Figure 21:**
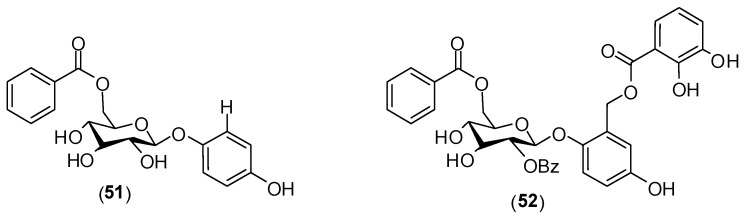
Phenolic glycosides **51** and **52**.

The screening of 850 ethyl acetate extracts of Madagascan plants was carried out resulting in the isolation of several phenolic glycosides from *Flacourtia ramontchi*. To assess antiviral activity, the authors conducted enzyme assays with purified enzyme NS5 polymerase of dengue virus. The substances **51** and **52** (Figure 21) were the most active phenolic derivatives evaluated against DENV NS5 polymerase. The observed activity was moderate and the determined IC_50_ values were 9.3 ± 2.8 μmol/L for **51** and 9.5 ± 5.0 μmol/L for **52**. The mechanism of action of these compounds needs to be further investigated [63].

**Figure 22:**
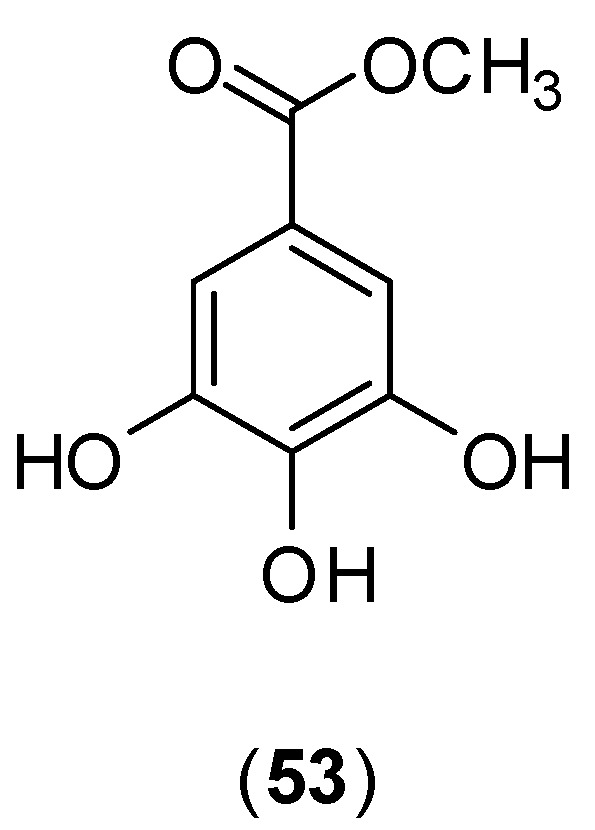
Structure of methyl gallate (**53**).

Rahman and collaborators conducted an *in vitro* experiment with C6/36 cells by MMT colorimetric assay against Dengue Virus serotype 2, and an enzyme assay with purified DENV-2 NS2B/3. Methyl gallate (**53**, Figure 22) which was purified from the methanol extract of *Quercus lusitanica* inhibited 98% of DENV-2 NS2B/3 protease at 0.3 mg/mL. Infected and treated C6/36 cells showed that the treatment with crude methanol extracts as well as methyl gallate (**53**) purified from the extract down-regulated the expression of the NS1 protein. This result could be related to a reduction or absence of a cytopathic effect on infected C6/36 cells [64].

## 8. Miscellaneous

**Figure 23:**
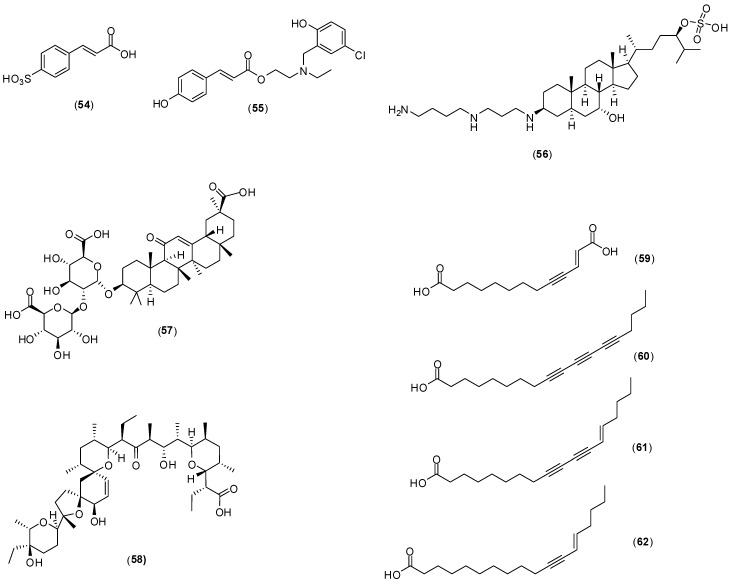
Structures of compounds **54**–**62**.

Several other compounds (Figure 23) have been reported to possess antivirus activity. Zoasteric acid (**54**) is obtained from the temperate marine eelgrass, *Zastera marina*. Rees and collaborators conducted *in vitro* experiments with LLCMK-2 cells by focus forming unit reduction assay and qRT-PCR to evaluate if compound **54** could inhibit all serotypes of dengue virus. Compound **54** presented a modest antiviral activity against serotype 2 with IC_50_ of approximately 2.3 mmol/L .This investigation also identified the synthetic analogue of zoasteric acid (**55**) as a more active compound concerning antidengue activity. It presented inhibitory effects against all DENV serotypes displaying IC_50_ of 24, 46, 14 and 47 μmol/L against DENV-1, DENV-2, DENV-3 and DENV-4, respectively. The analogue **55** showed support of inhibition at an entry step in the viral life cycle and enhanced virus-cell binding as evidenced by a quantitative RT-PCR assay system. The idea that compound **55** interferes with entry by promoting inappropriate virus-cell contacts would lend support to the hypothesis that these compounds function through binding to attachment domains on adherent organisms and subsequent release from the protected surface [65].

Squalamine (**56**) was first discovered in the tissues of the dog fish shark (*Squalus acanthias*) and later identified within the circulating white blood cells of the sea lamprey (*Petromyzon marinus*). The *in vitro* effect of squalamine (**56**) on dengue virus infection of human endothelial cells (HMEC-1) was evaluated by the plaque assay. At the concentration of 40 µg/mL, dengue infection was inhibited by 60%. The infection was completely suppressed at 100 µg/mL. The proposed mechanism of action involves the capacity of squalamine (**56**) to neutralize the negative electrostatic surface charge of intracellular membranes in a way that renders the cell less effective in supporting viral replication [66].

**Table 5:** Bioactivities determined for glycyrrhizin (**58**) against DENV-1-3.

Virus Serotype	EC_50_^a^ (μg/mL)	EC_90_^b^ (μg/mL)	Selectivity Index ^c^	
			Based on CC_50_ of Confluent Cells ^d^	Based on CC_50_ for Growing Cells ^e^
DENV-1	450.0	316	>6	6
DENV-2	174.2	317	>6	5
DENV-4	632.7	416	>6	4

^a^ Effective concentration required to reduce virus-induced cytopathogenicity by 50%; ^b^ Effective concentration required for inhibition of virus yield; ^c^ Seletivity index (CC_50_ divide by EC_50_); ^d^ Calculated from confluent cells, CC_50_ > 3000 μg/mL; ^e^ Calculated from exponentially growing cells, CC_50_ = 2500 μg/mL.

Crance and co-workers evaluated glycyrrhizin (**57**), the major component responsible for the sweet-tasting constituent of *Glycyrrhiza glabra* (liquorice) root, against eleven flaviviruses including DENV-1, DENV-2, and DENV-3 [67]. The antiviral evaluation was performed *in vitro* with Vero cells by plaque reduction assay. Several bioactivities were determined for this compound and they are summarized in Table 5. This antiviral compound has already been used in patients in the treatment of other diseases. It should be further considered for use, either alone or in combination with another antiviral compounds tested in this work (interferon, ribavirin, 6-azauridine) for the treatment of flavivirus infections.

Narasin (**58**) is a polyether antibiotic and antibacterial produced by fermentation of *Streptomyces aureofaciens*. A dose-dependent study revealed that **58** has a 50% inhibitory concentration of less than 1 μmol/L against all four DENV serotypes [68]. Minimal cytotoxicity was determined for this compound (50% cytotoxic concentration > 1,000 μmol/L). *In vitro* assays with Huh-7 cells by plaque assay with different concentrations, qRT-PCR, western blotting and ultrastructural imaging were performed in this investigation. Narasin (**58**) treatment of DENV-2 infected Huh-7 cells suggested that the compound is involved in inhibiting the post-entry stages of viral replication during DENV infection. The antiviral mechanism of narasin (**58**) is likely to be associated with the disruption of viral protein synthesis. No differences of RNA levels were found between narasin (**58**) treated and DENV-2 infected cells. The study indicated a characteristic disruption of viral protein synthesis by substance (**58**) without affecting viral RNA replication. However, a more detailed investigation is required to understand the exact molecular mechanism of narasin (**58**) in the inhibition of DENV protein synthesis and replication [68].

The acetylenic compounds **59**–**62** were isolated from an ethyl acetate extract of *Anacolosa pervilleana*, a Madagascan plant. By using purified DENV RdRp polymerase in enzyme assay, it was found that acetylenic compounds **59**–**62** give rise to IC_50_ values around 3 μmol/L in the DENV RdRp assay. The results show that compounds possess some selectivity toward DENV RdRp. All compounds except **59** showed an overall antimetabolic effect in Vero cells (CC50s between 20 and 30 μmol/L). The presence of an additional acidic group in compound **59**, probably prevents its penetration through the cell membrane, which may explain the absence of cytotoxicity [69].

## 9. Conclusions

During the last decades, the exploitation of the natural product pool has afforded a variety of compounds possessing activity against dengue virus serotypes. In several cases, very interesting activities are associated with the described compounds. It can be stated, though, that only a small fraction of the vast reservoir of compounds available from nature has been explored with the aim to find antivirus effective against dengue. From nature, it is possible that effective dengue antiviral compounds with low toxicity to human beings will certainly be found. Moreover, the structures of the natural compounds can serve as prototypes that can be optimized by synthetic campaigns in order to find even more active substances against dengue virus.

Most of the studies herein described were conducted *in vitro* using different plaque assays, including the focus forming assay, plaque reduction assay, plaque titration assay, virus yield reduction assay and MMT colorimetric assay. Some of these studies [38,39,47,49,50,54,56,57,64,65,68] sought to use more than one methodology to perform and/or verify the antiviral activity, giving greater strength to the results. Only three authors conducted *in vivo* studies [45,56], using animals, which further demonstrates the urgent need to make progress in these experiments to find a useful and effective antiviral against dengue. All of them were associated with *in vitro* assays. Seven enzyme assays, four against DENV NS5 polymerase [51,58,63,69] and three against DENV NS2B/NS3 protease [48,55,64] were conducted. The tests used are well established and are ideal for this type of research. It is also necessary to carry out more extensive studies both *in vitro* and *in vivo* followed by toxicity and clinical tests to further evaluate the potential of compounds obtained from nature as dengue antivirals. Finally, subsequent *in vivo* testing will indicate which of these substances are going to be the most promising in antiviral therapies against dengue, since efficiency *in vitro* does not necessarily have a parallel *in vivo*.
